# Can Knowledge of Genetic Distances, Genome Sizes and Chromosome Numbers Support Breeding Programs in Hardy Geraniums?

**DOI:** 10.3390/genes12050730

**Published:** 2021-05-13

**Authors:** Mehrdad Akbarzadeh, Katrijn Van Laere, Leen Leus, Jan De Riek, Johan Van Huylenbroeck, Stefaan P.O. Werbrouck, Emmy Dhooghe

**Affiliations:** 1 Plant Sciences Unit, Flanders Research Institute for Agricultural, Fisheries and Food Research (ILVO), Caritasstraat 39, 9090 Melle, Belgium; katrijn.vanlaere@ilvo.vlaanderen.be (K.V.L.); leen.leus@ilvo.vlaanderen.be (L.L.); jan.deriek@ilvo.vlaanderen.be (J.D.R.); johan.vanhuylenbroeck@ilvo.vlaanderen.be (J.V.H.); emmy.dhooghe@ilvo.vlaanderen.be (E.D.); 2 Department Plants and Crops, Faculty of Bioscience Engineering, Ghent University, Coupure Links 653, 9000 Ghent, Belgium; stefaan.werbrouck@ugent.be

**Keywords:** 2C value, AFLP, chromosome counts, cross compatibility, hybridization, Geraniaceae, hybrids, Jaccard similarity coefficient

## Abstract

Breeding programs in ornamentals can be facilitated by integrating knowledge of phylogenetic relatedness of potential parents along with other genomic information. Using AFLP, genetic distances were determined for 59 *Geranium* genotypes, comprising 55 commercial cultivars of the three subgenera of a total collection of 61 *Geranium* genotypes. A subgroup of 45 genotypes, including intragroup and intergroup hybrids, were selected and further characterized for genome sizes and chromosome numbers. The variation in genome size ranged from 1.51 ± 0.01 pg/2C to 12.94 ± 0.07 pg/2C. The chromosome numbers ranged from 26 to 108–110 with some hybrids showing an aberrant number of chromosomes based on their parents’ constitution. All chromosome numbers of *Geranium* are an even number, which presumes that unreduced gametes occur in some cross combinations. Overall, parental difference in genome size and chromosome number were not limiting for cross compatibility. Good crossing compatibility was correlated to a Jaccard similarity coefficient as parameter for parental relatedness of about 0.5. Additionally, parent combinations with high differences in the DNA/chromosome value could not result in a successful cross. We expect that our results will enable breeding programs to overcome crossing barriers and support further breeding initiatives.

## 1. Introduction

The Geraniaceae comprises the genera *Geranium* (430 species), *Pelargonium* (280 species), *Erodium* (80 species) and *Monsonia* (40 species) resulting in a total number of more than 700 species [1,2,3]. *Erodium* and *Geranium* are phylogenetically closest related [4]. Hardy *Geranium* or cranesbill is a genus divided into three subgenera: *Erodioidea* with 22 species in four sections, *Robertium* with 30 species in eight sections, and *Geranium* with 380 species in at least ten sections [5]. This division into sections is still in flux [6]. Yeo divided the sections further in taxonomical groups based on a key with morphological characteristics [7] (Figure S1).

*Geranium*, a cosmopolitan genus, is adapted to well-watered soils and cool temperate regions [7]. The majority of the species originate in the temperate northern part of Eurasia. Most sections of subgenus *Geranium* consist of perennials from the east Mediterranean region but also extend to the western Himalayas with many species found in South America as well [5,7,8]. The other two subgenera contain annuals, biennials and summer-dormant tuberous species. The main distribution area of subgenus *Erodioideae* is Mediterranean, whereas subgenus *Robertium* extends from Macaronesia to the Far East.

The cultivation of *Geranium* started very recently, from the 1990s onwards, with the plants being used as hardy perennials [7]. Together with the cultivation of the plant, breeding and the first interspecific crosses were initiated. The first cranesbills on the market were *G. macrorrhizum*, *G. sanguineum*, *G*. *himalayense*, *G. endressii* and *G.* × *oxonianum* (*G*. *endressii* × *G. versicolor*). Later, others appeared, such as *G. clarkei*, *G.* × *cantabrigiense* (*G. dalmaticum* × *G. macrorrhizum*) and ‘Ann Folkard’ which was obtained by crossing *G. procurrens* and *G. psilostemon*. The best-selling *Geranium* ever is *G.* ‘Rozanne’ which is characterized by a long flowering period, large flowers and vigorous growth [9]. This cultivar is obtained as a spontaneous cross between *G. himalayense* and *G. wallichianum* and is mass-produced by micropropagation. ‘Rozanne’ has been selected as Plant of the Century at the Royal Horticultural Society (RHS) Chelsea Flower Show’s 100th anniversary, received the Award of Garden Merit by RHS in 2006 and was honored as the Perennial Plant of the Year by the Perennial Plant Association (2008).

Most of the commercialized hardy geraniums are included in the subgenus *Geranium,* section *Geranium*. Until 2001, 165 different cross combinations had been released resulting from crosses between species in this section. In addition, 11, 14 and 13 different cross combinations between species of subgenus *Robertium*, within the subgenus *Erodioidea*, and between two subgenera (*Geranium* and *Erodioidea*), respectively, are on the market [7]. Although these different cross combinations have resulted in many commercial cultivars, not all cross combinations show equal success and many potentially interesting combinations are not possible at all. Specifically in the subgenus *Geranium, G. endressii* of the Endressii Group is compatible with the Sessiliflorum Group, while *G. nodosum* of this same Endressii Group is not compatible with any other species within this group [7] (Figure S1). Of the Sylvaticum Group, *G. sylvaticum* is crossable with *G. endressii* (Endressii Group) but only resulted in one hybrid with *G. pratense.* This combination, found in nature by Richard Nutt, could not be artificially reproduced [7]. Of this same Sylvaticum Group, *G. psilostemon* is the most successful genotype to cross with members of the Wallichianum Group and can be combined with *G. endressii* (Endressii Group), *G. pratense* (Pratense Group) and *G. sanguineum* (Sanguineum Group). *G. sanguineum* in turn can also be well combined with species of the Wallichianum Group. Members of the subgenus *Robertium* and members of the other two subgenera were found to be incompatible. All these examples clearly illustrate the limitations of the variation within *Geranium.* More knowledge about cross compatibility based on genome size, chromosome numbers and genetic relatedness would facilitate *Geranium* breeding.

Genome size is known to be an effective parameter for various plant characters like flower and shoot phenology. A positive correlation was seen between genome size and earliness of flowering as well as preferred environmental conditions in 219 geophytes [10,11]. Variation in genome size may also predict cross compatibility. For example, *Cirsium* species which are characterized by smaller genomes, tend to produce interspecific hybrids more frequently compared to those with larger genomes [12]. For the genus *Geranium*, genome sizes of some species from different taxonomic groups have been published, with variation between 1.44 and 8.36 pg/2C [13,14,15,16]. In addition, variation in chromosome numbers have been described [15,16,17,18]. For the subgenus *Geranium*, 11 chromosome numbers have been described [7]. The section *Geranium* has mostly 28 chromosomes (2n = 2x = 28). For some species, however, the chromosome number is higher, such as *G. macrorrhizum* (2n = 46) and *G. sanguineum* (2n = 84). The chromosome number 28 is also found in all sections of the subgenus *Erodioidea* and in three sections of subgenus *Robertium* [3,7]. Phylogenetic taxonomic studies have been performed for *Geranium* for species originating from specific areas as North [19] and South America [5], Hawaii [20], Turkey [21] and New Zealand [6]. Phylogeny and historical biogeography in some genera of Geraniaceae also have been studied [8,16,22]. However, much knowledge is still lacking about genome sizes, chromosome numbers and genetic relationships, both for wild types as well as commercially interesting species and genotypes.

Using data on phylogenetic relatedness, chromosome numbers, ploidy level and genome sizes of parental species, predictions can be made concerning success of hybridization, as many studies show [23,24]. Hybridization between parents with unequal chromosome numbers is either less likely to occur [25] and/or results in other ploidy levels or sterile progeny [26]. For example, in *Petunia* two groups of chromosome numbers (2n = 2x = 14 and 2n = 2x = 18) exist and crosses between these two groups are not yet possible [25]. A study on *Sarcococca* showed higher cross efficiency between parents with equal ploidy level and genome size compared to parents with different genome size and ploidy level, while far genetic distances were less of a barrier to hybridization [27]. Instead, for *Helleborus* only parents with a threshold of 0.264 or higher of genetic proximity could give rise to interspecific hybrid offspring [28]. For *Hydrangea*, it has been shown that hybrids between species with large phylogenetic distance are sterile or non-vigorous [29,30,31,32,33]. A study on *Buxus* resulted in interspecific hybrids after crosses between two genetically non-related parents (*B. sempervirens* (1.5 pg/2C) and *B. microphylla* (2.11–2.15 pg/2C)) [34]. In that study, AFLP analysis indicated that these hybrids were more closely related to *B. microphylla*, while their genome size was more similar to *B. sempervirens*. Many more examples are described in the literature.

In the present study, we examined the genome sizes, chromosome numbers and genetic relationships between different *Geranium* species and commercial cultivars including intra- and intergroup hybrids with the aim of supporting future breeding progams in *Geranium*. As our focus is on breeding we included mostly commercial cultivars. By combining our results with the available knowledge from the literature on cross compatibility in *Geranium,* we aim to provide thresholds for proximity values between parents for successful hybridizations. The knowledge obtained in this study about genetic relations, chromosome numbers, and genome sizes will elucidate the limits of variance in specific parameters and will contribute to successful combination of genotypes in breeding programs for *Geranium.*

## 2. Materials and Methods

### 2.1. Plant Material

A *Geranium* plant collection of 61 genotypes that covers all three subgenera of *Geranium* (*Geranium*, *Erodioidea* and *Robertium*) and includes 12 different taxonomical groups (with one genotype *G.* ‘Bob’s Blunder’ G03, with unknown parents) has been created at ILVO, Melle, Belgium (Table 1 and Table 2). The group division of Yeo [7] was used (Figure S1). We included 11 intragroup hybrids and 14 intergroup hybrids in this collection (Table 2). The plants were chosen based on whether they had morphological characteristics deemed valuable for commercial hardy geraniums. Plants were grown in 2L pots (peat-based substrate (Saniflor NPK 12:14:24, EC 45 mS/m)) except for *G. cinereum* which was potted in stone mixture (Kift) and were kept under greenhouse conditions (natural conditions, set-point for ventilation 10 °C). The identification of the specimens in the collection was confirmed by a collector of hardy geraniums (Dirk Gunst, Belgium).

### 2.2. AFLP 

DNA extraction was performed from 20 mg lyophilized young leaf material according to a modified CTAB DNA isolation protocol of Doyle and Doyle [35] for 59 genotypes of our *Geranium* collection. AFLP [36] was performed as in De Riek et al. [37] using the commercially available kit from Perkin-Elmer Biosystems for fluorescent fragment detection [36,37,38]. The primer combinations were: *EcoRI-*AAC/*MseI*-CAG, *EcoRI*-AAG/*MseI*-CAG, *EcoRI*-ACA/*MseI*-CTT and *EcoRI*-AGG/*MseI*-CAG. Band scoring and statistical analyses was based on De Riek et al. [37]. After calculating of a proximity matrix using the Jaccard similarity coefficient, both clustering and Principal Coordinate Analysis (PCoA) were applied. 

### 2.3. Genome Sizes

Genome sizes were analyzed on young leaf samples on a Quantum P flow cytometer equipped with a laser (488 nm; 180 mW) and CyPAD software (Quantum Analysis, Münster, Germany). The CyStain PI kit (Sysmex, Münster, Germany) was used for sample preparation. One piece (±0.5 cm²) of fresh young leaf tissue was chopped using a razor blade [39] together with fresh leaf tissue of the internal standard in 0.4 mL of extraction buffer. Samples were filtrated through a 50 μm CellTrics filter (Sysmex, Münster, Germany) and subsequently 1.2 mL staining solution (CyStain PI kit) with propidium iodide and RNase A stock solution (both CyStain PI kit) were added. To improve the quality of the histograms, 1.25% of polyvinylpyrrolidone (PVP10) (Duchefa, Haarlem, The Netherlands) was added to the staining buffer as it is known to bind phenolic compounds [40]. Samples were incubated in the dark at 4 °C for at least 30 min before measurement. For each genotype three samples were prepared. These three samples were prepared on different days and, when possible, samples were taken from different plants of the genotype. Each sample analyzed using the flow cytometer histograms obtained with the FL2 and FL3 detector was also analyzed using CyPAD software (Quantum Analysis, Münster, Germany). Genome sizes were calculated from the ratios between peak positions of the *Geranium* sample and the internal reference with known genome size. Mean values and standard deviations were calculated based on at least six histograms obtained (three samples, two histograms per analysis). Due to the wide range of genome sizes in the genus *Geranium*, different internal standards were used: *Pisum sativum* ‘Ctirad’ 9.09 pg/2C [41], *Zea mays* ‘CE-777’ 5.43 pg/2C [42] and *Glycine max* ‘Polanka’ 2.50 pg/2C [43]. Terminology on 2C values is used as defined by Greilhuber et al. [44]. A calculated value for the DNA content per chromosome was determined by dividing the genome size through the chromosome number (see below). 

### 2.4. Chromosome Numbers

Young root tips were harvested from 2-week-old cuttings of each genotype. The root tips were pre-treated with 0.1% colchicine + 0.2% 500× 8-hydroxyquinoline during 3 h at 4 °C. Then, the root tips were fixed in 3:1 EtOH:acetic acid for 60 min at room temperature. Cell suspensions and slide preparation was done according the “SteamDrop” protocol [45]. A 0.6% enzyme solution (0.6% cellulase, 0.6% pectolyase, and 0.6% cytohelicase in 0.1 M citrate buffer (0.1 M sodium citrate tribasic dihydrate + 0.1 M citric acid)) was used for incubation at 37 °C for 40 min. Fixatives used during slide preparation were 2:1 and 1:1 EtOH:acetic acid. Chromosome slides were stained with 1% DAPI (100 µg/mL) diluted in Vectashield and analyzed using a fluorescence microscope (AxioImager M2, Carl Zeiss MicroImaging, Belgium) equipped with an Axiocam MRm camera and ZEN software (Carl Zeiss MicroImaging, Belgium) at magnification 1000×. Image analysis was performed in DRAWID v0.26 [46] on at least five well-spread metaphases of each genotype.

## 3. Results

**Figure 1:**
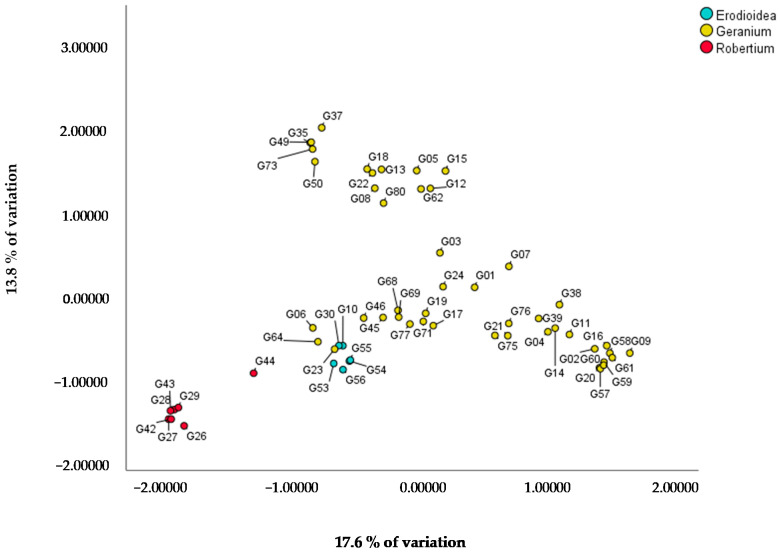
PCoA based on AFLP data of 61 genotypes of *Geranium* with the X- and Y-axis comprising 17.6% and 13.8% of the variation, respectively.

AFLP analysis with four primer pairs generated a total dataset of 872 markers. Based on a PCoA with two factors explaining 17.6% and 13.8% of the variation, a division in the subgenera of the *Geranium* genus is given (Figure 1): *Geranium* and *Erodioidea* subgenera are more closely related compared to the subgenus *Robertium*. Of the five analyzed genotypes in subgenus *Robertium*, only G44 (*G. macrorrhizum* ‘White Ness’) is closer to the *Geranium* and *Erodioidea* subgenera.

**Figure 2:**
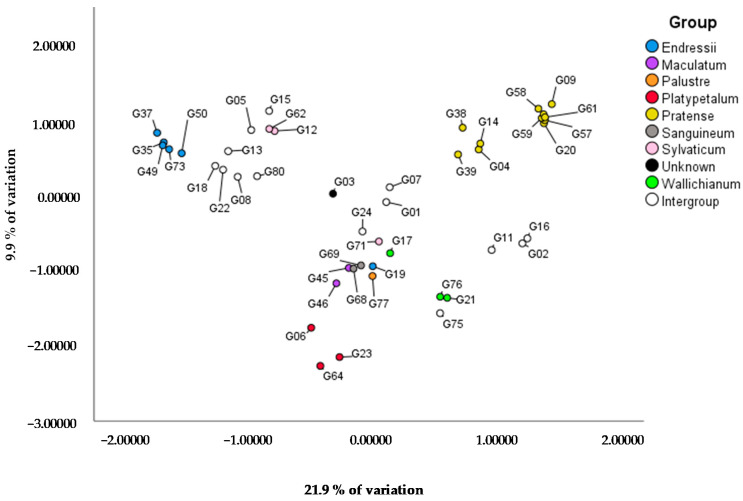
PCoA based on AFLP data of genotypes of subgenus *Geranium* (in total 45 plants) with X and Y-axis explaining 21.9% and 9.9% of the variation, respectively.

When specifically focusing on the subgenus *Geranium*, it can be concluded that the genotypes within a taxonomical group mostly cluster in a two-factorial PCoA explaining 21.9% and 9.9% of the variation (Figure 2). The highest genetic distance is found between the Pratense, Platypetalum and Endressii Group, with Platypetalum being the only group in the *Tuberosa* section. The Endressii Group has one outlier (G19; *G. nodosum* ‘Silverwood’). G71 (*G. sylvaticum* ‘Album’) is separated from the other two Sylvaticum Group genotypes (G12 and G62), representing *G. psilostemon*. This division at species level is also visualized in the Pratense Group and Wallichianum Group. For the Pratense Group, three clusters can be recognized: (1) only genotypes of *G. pratense* (G09, G20, G57, G58, G59 and G60), (2) the ‘Brookside’ cultivar G04 and *G.* ‘Orion’ G14 (which has ‘Brookside’ as a parent), which are intragroup crosses with *G*. *pratense* as one parent, and (3) G38 and G39, which are both cultivars of *G. himalayense*. In the case for the Wallichianum Group, the intragroup cross G17 (*G.* ‘Salome’) is separated from the *G. wallichianum* species G21 and G76.

Almost all intergroup hybrids are clearly located between the six parent groups (Figure 2). For example, most of the intergroup hybrids between the Pratense Group and the Wallichianum Group (G02, G11, G16 and G75) are found in between these two groups, except for G75 which is located more closely to the Wallichianum-type genotypes. G01 (*G.* ‘Anne Thomson’) and G07 (*G.* ‘Dragon Heart’) which are progeny of crosses between *G. procurrens* (Wallichianum Group) and *G. psilostemon* (Sylvaticum Group) are located in between their parents’ groups. The intergroup hybrids between the Endressii Group and the Sessiliflorum Group G08, G13, G18 and G22 are all clustered together. G24 (*G.* ‘Tiny Monster’), progeny of the intergroup combination between *G. psilostemon* and *G. sanguineum*, is genetically closer to the included *G. sanguineum* genotypes (G68 and G69) than to the *psilostemon* genotypes (G12 and G62) of this collection. G15 (*G.* ‘Patricia’), an intergroup hybrid between the Endressii Group and the Sylvaticum Group (G. *psilostemon* types), is more closely related to the G. *psilostemon* types than to the Endressii Group. For G05 (*G.* ‘Catherine Deneuve’), the literature does not agree whether this is a cross product of *G. psilostemon* and *G.* × *oxonianum* or *G. psilostemon* and *G. procurrens*. Our PCoA does not indicate close relation to the *G. psilostemon* × *G. procurrens* cross products from our collection (G01 and G07). 

**Figure 3:**
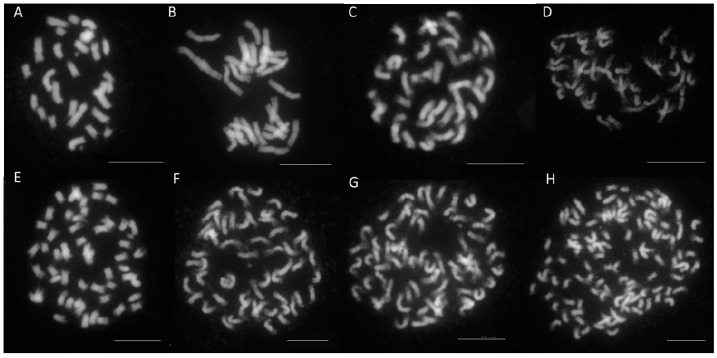
Chromosome counts of *Geranium* genotypes: (**A**) *G. phaeum* ‘Angelina’—3.52 pg/2C and 2n = 28; (**B**) *G. wallichianum* ‘Sylvia’s surprise’—6.78 pg/2C and 2n = 38; (**C**) *G.* × *riversleaianum* ‘Mavis Simpson’—4.31 pg/2C and 2n = 38; (**D**) *G.* ‘Azure Rush’—8.90 pg/2C and 2n = 42; (**E**) G. ‘Catherine Deneuve’—6.95 pg/2C and 2n = 54; (**F**) *G. himalayense* ‘Derrick Cook’—7.49 pg/2C and 2n = 56; (**G**) *G. sanguineum* ‘Blushing Turtle’—7.80 pg/2C and 2n = 66; (**H**) *G.* ‘Tiny Monster’—12.94 pg/2C and 2n = 108/110 (bar = 10 µm).

Table 1 and Table 2 present the genome sizes, chromosome numbers and DNA content per chromosome of a subgroup of 45 *Geranium* genotypes. The dendrogram of these genotypes is depicted in supplemental Figure S2. The genome sizes, chromosome numbers and calculated DNA content per chromosome within a taxonomical group and certainly within a species are mostly similar for the subgenera *Geranium* and *Robertium*, with only certain intergroup crosses as exception (Table 1 and Table 2, Figure S3). However, between groups large differences do occur. For example, the Pratense, Wallichianum, Platypetalum, Cinereum and Phaeum Groups all have 28 chromosomes but a different genome size (Figure S3, Figure 3). 

The Macrorrhizum Group, with analyzed species of *G. macrorrhizum* G42 (1.60 ± 0.02 pg/2C), *G.* × *cantabrigiense* G27 (1.51 ± 0.01 pg/2C) and *G. dalmaticum* G82 (1.56 ± 0.06 pg/2C), has the lowest genome sizes with high chromosome numbers (2n = 2x = 46; G82 not measured). Consequently, the DNA content per chromosome of the Macrorrhizum Group is low, around 0.030 (Table 1 and Table 2, Figure S3c). In G44 (*G. macrorrhizum* ‘White Ness’), also belonging to the Macrorrhizum Group of the subgenus *Robertium*, the genome size (2.96 ± 0.06 pg/2C) and chromosome number (2n = 4x = 92) is doubled in comparison to other members of the Macrorrhizum Group, confirming tetraploidy for this genotype (Table 1, Figure S3). In the Pratense Group, the separation that was observed between *G. pratense* species and *G. himalayense* species based on PCoA (Figure 2) was confirmed by the presence of two groups with differing genome sizes: one comprised of G14, G38, G39 (6.93 ± 0.06, 7.31 ± 0.08, 7.49 ± 0.09 pg/ 2C, respectively), and another comprised of G04, G09, G57, G61 and G81 (4.54 ± 0.0, 4.90 ± 0.03, 5.08 ± 0.04, 5.08 ± 0.02 and 4.70 ± 0.07 pg/2C, respectively) (Table 1 and Table 2, Figures S2 and S3a ). The higher genome size group has a chromosome number of either 42 or 56, while genotypes from the lower genome size group only have 28 chromosomes (Table 1 and Table 2, Figure S3b). 

The DNA content per chromosome of the selected collection is mostly between 0.103 and 0.185 pg/chromosome except for Wallichianum, Palustre, Macrorrhizum and Cinereum Groups (Table 1 and Table 2, Figure S2). For Wallichianum, Palustre and Cinereum this value was higher than 0.200 pg/chromosome. Species in the Wallichianum group and Cinereum group have rather high genome sizes (between 6.06 and 6.86 pg/2C) and only 28 chromosomes. The highest genome sizes of the collection, excluding intergroup hybrids, were seen in G77 (*G. wlassovianum*, Palustre Group) and G69 (*G. sanguineum* ‘Album’, Sanguineum Group) with 11.50 ± 0.02 and 8.78 ± 0.07, respectively (Table 1 and Table 2, Figure S3a). For G69, this high genome size is linked with a high chromosome number of 2n = 84, while G77 has a chromosome number of 56 (Table 1, Figure S3b).

The intra- and intergroup hybrids reveal different scenarios according to chromosome count (Table 2, Figure 3). For the (generally more easily obtained) intragroup hybrids, the chromosome number examined in the hybrids can be predicted by the sum of the gametes of the parental species. This is also the case for the intergroup hybrid *G.* ‘Rozanne’ G16. However, for most of the intergroup hybrids an aberrant chromosome number was counted, assuming unreduced gametes could be involved (Table 2, Figure 3). For example, *G.* ‘Catherine Deneuve’ G05 with 54 chromosomes could be a result of the unreduced number of *G. psilostemon* and the normal gamete constitution of *G.* × *oxonianum*, which makes 55 chromosomes. For G. ‘Bloomtime’ G75, a reduction in chromosome number occurred compared to its parental chromosome constitution.

The genome size of hybrids as compared to their parents (Table 2) were mostly in line with expectations based on parental genome size. Exceptions were the intergroup crosses G05, G16 and G24, as these crosses showed a larger genome size value than expected. For G05 and G24, a possible explanation could be the occurrence of unreduced gametes in one or both parents. The DNA content per chromosome of intra- or intergroup hybrids showed no deviation compared to its parents (Table 2). 

In Table 3 the Jaccard similarity coefficients of the parental species and cultivars of described hybrids is shown. Jaccard similarity coefficients in our collection range between 0.091 between *G. macrorrhizum* and *G. pratense* (different groups within subgenus *Geranium*) and 0.506 between *G. endressii* and *G. versicolor* (both in Endressii group). The nearest Jaccard similarity coefficients between two subgenera is 0.164, i.e., between *G*. *cinereum* (in Cinereum Group of subgenus *Erodioidea*) and *G*. *maculatum* (in Maculatum Group of subgenus *Geranium*). An examination of Jaccard similarity coefficients of either existing interspecific crosses included in our collection or known cross combinations in the literature reveals that the lowest Jaccard similarity coefficient found in an intergroup hybrid is 0.130 (*G.* ‘Salome’ × *G.* × *oxonianum*). We therefore presume this could be a possible theoretical threshold for cross-compatibility in *Geranium*.

## 4. Discussion

The PCoA of all the plants of our collection was in accordance with Yeo [7] who divided the *Geranium* genus into these three subgenera based on different seed discharge mechanisms [47]. In addition, as also discovered by Marcussen et al. [8], the subgenera *Geranium* and *Erodioidea* clustered more closely together compared to *Robertium*. This was already hypothesized by Bremner, a *Geranium* breeder who could not achieve any successful cross between species of subgenus *Robertium* and the two other subgenera [7]. Based on a similar PCoA performed specifically for the *Geranium* subgenus, the Pratense, Platypetalum and Endressii Groups showed the highest genetic distance in our collection, with the Platypetalum Group being the only one in the *Tuberosa* section and also clearly isolated from the other groups in the *Geranium* section [7]. Bremner also noted that no viable seeds were obtained in crosses between Platypetalum and other groups and no offspring is yet described between species of the three most genetically distant groups: Pratense, Platypetalum and Endressii [7]. Our AFLP analysis reveals a possible error in the classification into taxonomic groups by Yeo [7]. In our opinion, *G. nodosum* (G19) is erroneously classified in the Endressii Group as it is not clustered with other genotypes of the Endressii Group and its closest relative in our collection is *G. wlassovianum* (G77) in the Palustre Group. This could explain the failure of intragroup crosses using *G*. *nodosum* [7]. The chromosome counts found in our study are in accordance with the literature [14,17,48,49,50,51,52,53,54,55]. Our results for *G. wallichianum* G21 and G76 (2n = 2x = 28) were confirmed by Kumar et al. [49,51] although some studies report 2n = 2x = 26 [56] and 2n = 4x = 56 [48]. As Yeo reports [17], 2n = 2x = 28 is most common in *Geranium* genotypes. Our study reports chromosome numbers for an additional 19 genotypes, mostly commercial cultivars, for which no chromosome data were previously available. This results in 12 chromosome numbers in the section *Geranium* instead of 11 as reported by Yeo [7]. The genome sizes found in our collection were within the range of those reported in the literature [15,16,57,58]. The genome sizes found by Zonneveld [16] for *G. endressii* (3.13 pg/2C), *G. nodosum* (3.53 pg/2C), *G. phaeum* (3.37 pg/2C), *G. pratense* (4.83 pg/2C), *G. sanguineum* (8.36 pg/2C) and *G. sylvaticum* (4.92 pg/2C) were also in the same range as in our study. The only difference between our results and the literature was the genome size of *G. macrorrhizum* (G42) (1.60 pg/2C in our study versus 3.71 pg/2C in the literature) [14]. Drawing conclusions on polyploidy is not easy as variation in ploidy levels was seldom found within a species. For *G. macrorrhizum* ‘White Ness’ G44 (2n = 4x = 92) tetraploidy was noticed, as confirmed by Tan [15], while G42, *G. macrorrhizum* ‘Czakor’, is assumed to be diploid (2n = 2x = 46). Tetraploids are rare, but for *G. maccrorhizum* and other members of section *Ruberta*, polyploidy and amphidiploidy are clearly evident [17]. Our observations on genome size and ploidy level of *G. macrorrhizum* were confirmed by Kato et al. [58] who also reported a hexaploid population in the Croatia population (2n = 6x = 3.77 pg/2C).

When analyzing the intergroup hybrids, our AFLP analysis, together with the results from chromosome counts and genome size measurements, was able to show their genetic background. A hypothesis is that the DNA content per chromosome in a hybrid seems to resemble the relatedness of the hybrid to its parent; when this value was more similar to one of the parents, this genotype also appeared to be more closely related to this parent in the AFLP. This indicates that the calculated DNA content per chromosome in *Geranium* can be valuable for predicting genetic relatedness without an AFLP analysis. For example, the successful hybrid cultivar ‘Rozanne’ (G16), together with the other crossing products of *G. wallichianum* and *G. himalayense* (G02, G11 and G75) are more closely related to the *G. wallichianum* parent. *G.* ‘Rozanne’ (G16) and *G.* ‘Azure Rush’ (G02) have 42 chromosomes, while *G.* ‘Bloomtime’ (G75) has 2n = 2x = 28. However, all three hybrids have a similar DNA content per chromosome of about 0.22 to 0.26 pg/chromosome, comparable to the *G*. *wallichianum* parent. For *G.* ‘Orion’ (G14), the presence of 42 chromosomes clearly reveals its background of a cross between *G.* ‘Brookside’ with 28 chromosomes and *G. himalayense* with 56 chromosomes, although its DNA content per chromosome is more similar to the ‘Brookside’ parent. Observations for *G.* ‘Blushing Turtle’ (G80) showed a clustering near its one parent *G. sanguineum*, but its chromosome number showed its intergroup character with an aberrant chromosome number of 2n = 66. *G.* ‘Blushing Turtle’ is a crossing product between *G. sanguineum* with 84 chromosomes [16] and *G.* × *oxonianum* (with 26 chromosomes in our collection) or *G. asphodeloides* (US Plant Patent 22376). *G. asphodeloides* has 24 chromosomes [2,23]. By calculating all scenarios based on the chromosomal characteristics of the parents, a possible hypothesis is that *G.* ‘Blushing Turtle’ contains all the chromosomes of *G. asphodeloides* as well as the haploid level of *G. sanguineum*, resulting in 66 chromosomes. The origin of *G.* ‘Bob’s Blunder’ (G03) remains unknown. In our AFLP analysis G03 has the highest relatedness with the intergroup hybrids between the Endressii Group and the Sessiliflorum Group (e.g., G18). Additionally, these hybrids are characterized by brown-colored leaves, which is also the case for ‘Bob’s Blunder’. Furthermore, it has a similar chromosome number of 2n = 38. Our AFLP was also able to uncover a possible parent for genotype G05, G. ‘Catherine Deneuve’, whose parentage was in doubt. There are two hypotheses about the parentage of G05; US Plant Patent license 223370 [59] described that parents are *G. psilostemon* and *G. procurrens,* while later the breeder expressed the belief that it was a hybrid between *G. psilostemon* and *G.* × *oxonianum*. In the dendrogram of our study, G05 is more closely related to to *G.* ‘Patricia’ (G15), a hybrid between *G. endressii* and *G. psilostemon.* As *G. endressii* is one of the parents of *G.* × *oxonianum*, we presume the breeders’ opinion is correct. The chromosome number of G05 (2n = 54) could not be explained by the normal chromosome contribution of the parents (*G.* × *oxonianum* 26 chromosomes and *G. psilostemon* 42 chromosomes); however, when the unreduced chromosome complement of *G. psilostemon* is combined with the reduced number of *G.* × *oxonianum* (13), the sum is almost correct. *G*. ‘Tiny Monster’ (G24) showed the highest chromosome number (2n = 108−110). Presuming the absence of unreduced gametes, this is difficult to explain based on the chromosome number of the parents (*G. sanguineum* 2n = 84 and *G*. *psilostemon* 2n = 42). Additionally, the intergroup hybrids of Endressii and Sessiliflorum Group, like *G.* ‘Sanne’ (G18), *G.* ‘Tanya Rendall’ (G22) and *G.* × *riversleaianum* ‘Mavis Simpson’ (G13) which are crosses between (1) *G. sessiliflorum* (2n = 52) [56] and *G.* × *oxonianum* (2n = 2x = 26, this study), (2) *G.* × *antipodeum* (no chromosome data but cross between *G. sessiliflorum* and *G. traversii*) and *G.* × *oxonianum* (2n = 2x = 26, this study) and (3) *G. endressii* (2n = 2x = 26 or 28, this study) and *G. traversii* (2n = 2x = 52−54) [56,60], respectively, all show a similar chromosome number of 2n = 2x = 38, which is clearly the result of hybridization. This study showed that chromosome numbers, genome sizes, genetic relatedness and DNA content per chromosome could provide additional information about genotypes with unknown or unclear parentage. The examination of hybrids in this study also revealed that similar chromosome number is not a prerequisite for successful cross combinations, although these successful hybrids often result in aberrant chromosome numbers. An aberrant chromosome number is often found in interspecific crosses and occurs more in genetically distant cross combinations [61]. A high level of variance in parental genome sizes and DNA content per chromosome was not observed in many intra- and intergroup hybrids included in this study, indicating that similarity of genome size and DNA content could be a requirement for successful hybridization. *G. nodosum* cannot be crossed with *G. endressii* despite similar chromosome numbers, similar genome size and similar DNA content per chromosome. Their pairwise proximity coefficient is 0.173, which presumably indicates the importance of the genetic distance in successful mating, as we have observed in *Geranium.* This study has shown that the presumed lower limit of 0.130 of pairwise Jaccard similarity coefficient of the parents can be taken as a threshold to successful intergroup crosses in the *Geranium* genus (without taking all other breeding barriers into consideration), with the value around 0.5 indicating easier (intragroup) crossings. An examination of the pairwise genetic proximity of the species of *Robertium* (*G.* × *cantabrigiense* and *G. macrorrhizum*) in comparison with the species of the other subgenera in this collection revealed many values below this 0.130 limit. The maximum Jaccard similarity coefficient between them was 0.151 (between *G.* × *cantabrigiense* and *G. endressii*) and the minimum was 0.091 (between *G. pratense* and *G. macrorrhizum* or *G.* × *cantabrigiense*). This could explain the inability of *Robertium* species to cross with other subgenera. In addition, the DNA content per chromosome (approximately 0.03) in the subgenus *Robertium* was clearly atypical in comparison to all other genotypes, which raises the hypothesis that also this could affect success of hybridization with other subgenera and species. 

## 5. Conclusions

We can conclude that knowledge about the genetic distance, genome size or chromosome number can support breeding efforts in *Geranium*. The DNA content per chromosome can be used as an alternative method to calculate the relatedness of the hybrid with its parents. Clearly, genetic relatedness is very important for cross compatibility in *Geranium*. We showed that easy cross combinations mostly have a pairwise proximity distance of around 0.5, with a minimum value of 0.130 required for hybridization. Although other fertilization barriers can probably also limit crossing success, we hypothesize that the pairwise Jaccard similarity coefficients can be a possible first determinator for successful cross hybridization in *Geranium.* An analysis of crossing products showed that an equal number of chromosomes is not a prerequisite to result in successful offspring and chromosome numbers are often aberrant in intergroup hybrids. As regards there being no odd chromosome numbers in *Geranium*, perhaps abnormal gametogenesis, like unreduced gamete production, in *Geranium* is common which should be evaluated in a future study. Genome sizes vary widely in *Geranium*, but successful crosses often show a more narrow variation in genome size between the parents. 

When working with as-yet unknown genotypes, breeders can use our data and measurements of genome size and chromosome numbers to gain information about the plants’ backgrounds and predict the success of potential crossings. This will support the success of future breeding programs.

## Figures and Tables

**Table 1:** List of all commercial cultivars and wild genotypes per subgenus included in the collection of Geranium and their genome sizes (Gen. size (pg/2C)), chromosome numbers (Chrom. numb.) and DNA/chromosome (DNA/chrom. (pg/chromosome)) (divided into taxonomical groups according to Yeo [7]). The plant species used as internal standard is mentioned with the genome size (*Zea mays* (M), *Pisum sativum* (P) and *Glycine max* (S)). The abbreviations for the taxonomical groups are the following: C = Cinereum, E = Endressii, M = Maculatum, MA = Macrorrhizum, P = Phaeum, PA = Palustre, PL = Platypetalum, PR = Pratense, SA = Sanguineum, SE = Sessiliflorum, SY = Sylvaticum, W = Wallichianum.

Genotype Number	Name	Taxonomical Group	Gen. Size (pg/2C)	Chrom. Numb.	DNA/Chrom.
**Subgenus *Geranium***					
G03	*G.* ‘Bob’s Blunder’ ^y^	Unknown	**5.31 (S)**	2n = **38**	**0.140**
G09	G. *pratense* ‘Galactic’ ^y^	PR	**4.92 (P)**	2n = **28**	**0.175**
G12 ^z^	*G. psilostemon* ‘Matu Vu’ ^y^	SY	-	-	-
G19	*G. nodosum* ‘Silverwood’ ^y^	E	**3.38 (M)**	2n = **28**	**0.121**
G20 ^z^	*G. pratense* ‘Summer Skies’ ^x^	PR	-	-	-
G21	*G. wallichianum* ‘Sylvia’s Surprise’^y^	W	**6.78 (P)**	2n = **28**	**0.242**
G35	*G. endressii* ^x^	E	**3.12 (M)**	2n = **26**	**0.120**
G37	*G. endressii* ‘Trevor Bath’ ^x^	E	**3.48 (M)**	2n = **28**	**0.124**
G38	*G. himalayense* ‘Baby Blue’ ^x^	PR	**7.31 (M)**	2n = **56**	**0.130**
G39	*G. himalayense* ‘Derrick Cook’ ^x^	PR	**7.49 (M)**	2n = **56**	**0.134**
G45	*G. maculatum* ‘Album’ ^x^	M	**7.64 (M)**	2n = **52**	**0.147**
G46	*G. maculatum* ‘Elizabeth Ann’ ^x^	M	**7.63 (M)**	2n = **52**	**0.147**
G57	*G. pratense* ‘Algera Double’ ^y^	PR	**5.08 (P)**	2n = **28**	**0.185**
G58 ^z^	*G. pratense* ‘New Dimension’ ^w^	PR	-	-	-
G59 ^z^	*G. pratense* ’Cloud Nine’ ^w^	PR	-	-	-
G60 ^z^	*G. pratense* ‘Plenum Album’ ^w^	PR	-	-	-
G61	*G. pratense* ‘Purple Ghost’ ^w^	PR	**5.08 (P)**	2n = **28**	**0.181**
G62	*G. psilostemon* ^y^	SY	**5.18 (P)**	2n = **42**	**0.123**
G64	*G. renardii* ^x^	PL	**2.35 (M)**	2n = **28**	**0.084**
G68 ^z^	*G. sanguineum* ‘Pink Pouffe’ ^x^	SA	-	-	-
G69	*G. sanguineum* ‘Album’ ^x^	SA	**8.78 (M)**	2n = **84**	**0.104**
G71	*G. sylvaticum* ‘Album’ ^w^	SY	**4.76 (P)**	2n = **28**	**0.170**
G73	*G. versicolor* ^w^	E	**3.53 (M)**	2n = **26/28**	**0.126–0.136**
G76	*G. wallichianum* ‘Havana Blue’ ^x^	W	**6.86 (P)**	2n = **28**	**0.245**
G77	*G. wlassovianum* ^x^	PA	**11.50 (P)**	2n = **56**	**0.205**
G81	*G. clarkei* ‘Kashmir Purple’ ^x^	PR	**4.70 (P)**	2n = **28**	**0.168**
**Subgenus *Erodioidea***					
G10	*G. cinereum* ‘Jolly Jewel Red’ ^x^	C	**6.39 (P)**	-	-
G30	*G. cinereum* ‘Laurence Flatman’ ^w^	C	**6.06 (P)**	2n = **28**	**0.217**
G53 ^z^	*G. phaeum* ‘Album’ ^x^	P	-	-	-
G54	*G. phaeum* ‘Angelina’ ^x^	P	**3.52 (M)**	2n = **28**	**0.126**
G55 ^z^	*G. phaeum* ‘Blauwvoet’ ^x^	P	-	-	-
G56 ^z^	*G. phaeum* ‘Klepper’ ^x^	P	-	-	-
**Subgenus *Robertium***					
G42	*G. macrorrhizum* ’Czakor’ ^y^	MA	**1.60 (M)**	2n = **46**	**0.035**
G43 ^z^	*G. macrorrhizum* ‘Spessart’ ^w^	MA	-	-	-
G44	*G. macrorrhizum* ‘White Ness’ ^x^	MA	**2.96 (M)**	2n = **92**	**0.032**
G82	*G. dalmaticum* ^x^	MA	**1.56 (S)**	-	-

^z^ These genotypes are not further characterized for genome sizes and chromosome numbers. Source of plant material with ^y^ Denis-Plants, ^x^ Jan Spruyt—Van der Jeugd, ^w^ Kwekerij Jan Neelen.

**Table 2:** Intra- and intergroup hybrids together with parental background and the hybrids’ respective genome sizes (Gen. size (pg/2C)), chromosome numbers (Chrom. numb.) and DNA/chromosome (DNA/chrom. (pg/chromosome)). The plant species used as internal standard is mentioned with the genome size (*Zea mays* (M), Pisum sativum (P) and *Glycine max* (S)). The figures in bold were obtained from our study; the others mentioned come from the literature. The abbreviations for the taxonomical groups are the following: C = Cinereum, E = Endressii, M = Maculatum, MA = Macrorrhizum, P = Phaeum, PA = Palustre, PL = Platypetalum, PR = Pratense, SA = Sanguineum, SE = Sessiliflorum, SY = Sylvaticum, W = Wallichianum.

**Subgenus *Geranium-*Ntragroup Crosses**												
**Genotype Number**	**Name**	**Gen. Size (pg/2C)**	**Chrom. Numb. (2n = )**	**DNA/Chrom.**	**Parent 1**	**Gen. Size (pg/2C)**	**Chrom. Numb. (2n = )**	**DNA/Chrom.**	**Parent 2**	**Gen. Size (pg/2C)**	**Chrom. Numb. (2n = )**	**DNA/Chrom.**
G04	*G.* ‘Brookside’ ^w^	**4.54 (M)**	**28**	**0.162**	*G. pratense* (PR)	**4.92–5.08**, 4.83	**28**, 28, 56	**0.175–0.185**	*G. clarkei* (PR)	**4.70**	**28**	**0.168**
G06	*G.* ‘Chantilly’ ^w^	**2.31 (M)**	**28**	**0.082**	*G. gracile* (PL)	-	-	-	*G. renardii* (PL)	**2.35**	**28**	**0.084**
G14	*G.* ‘Orion’ ^w^	**6.93 (P)**	**42**	**0.165**	*G.*‘Brookside’ (PR)	**4.54**	**28**	**0.162**	*G. himalayense* (PR)	**7.31–7.49**	**56**	**0.130–0.134**
G17	*G.* ‘Salome’ ^w^	**9.25 (S)**	-	-	*G. lambertii* (W)	-	-	-	*G. procurrens* (W)	-	-	-
G23 ^z^	*G.* ‘Terre Franche’ ^v^	-	-	-	*G.*‘Philippe Vapelle’ (PL)	-	-	-	*G. platypetalum* (PL)	-	-	-
G49	*G.* × *oxonianum* ‘Katherine Adele’ ^w^	**3.73 (M)**	**26**	**0.143**	*G. versicolor* (E)	**3.53**	**26/28**	**0.126–0.136**	*G. endressii* (E)	**3.12–3.48**, 3.13	**26/28**, 26, 28	**0.120–0.124**
G50	*G.* × *oxonianum* ‘Southcombe Double’ ^w^	**3.22 (P)**	**26**	**0.124**	*G. versicolor* (E)	**3.53**	**26/28**	**0.126–0.136**	*G. endressii* (E)	**3.12–3.48**, 3.13	**26/28**, 26, 28	**0.120–0.124**
**Subgenus *Geranium-*Intergroup Crosses**												
**Genotype Number**	**Name**	**Gen. Size (pg/2C)**	**Chrom. numb. (2n = )**	**DNA/chrom.**	**Parent 1**	**Gen. Size (pg/2C)**	**Chrom. numb. (2n = )**	**DNA/chrom.**	**Parent 2**	**Gen. Size (pg/2C)**	**Chrom. numb. (2n = )**	**DNA/chrom.**
G01	*G.* ‘Anne Thomson’ ^x^	**8.27 (M)**	**58**	**0.143**	*G. procurrens* (W)	-	-	-	*G. psilostemon* (SY)	**5.18**	**42**	**0.123**
G02	*G.* ‘Azure Rush’ ^x^	**8.90 (M)**	**42**	**0.212**	*G. wallichianum* (W)	**6.78–6.86**	**28**, 26, 28, 56	**0.242–0.245**	G. ‘Rozanne’ (W × PR)	**9.25**	**42**	**0.220**
G05	*G.* ‘Catherine Deneuve’ ^w^	**6.95 (M)**	**54**	**0.129**	*G. psilostemon* (SY)	**5.18**	**42**	**0.123**	*G.* × *oxonianum* (E) or *G. procurrens* (W)	**3.22–3.73** or -	**26** or -	**0.124–0.143** or -
G07	*G.* ‘Dragon Heart’ ^w^	**12.57 (P)**	**88**	**0.143**	*G. psilostemon* (SY)	**5.18**	**42**	**0.123**	*G. procurrens* (W)	-	-	-
G08 ^z^	*G.* ‘Dusky Crûg’ ^v^	-	-	-	*G.* × *antipodeum*^y^ (SE)	-	-	-	*G.* × *oxonianum* (E)	-	-	**0.124–0.143**
G11 ^z^	*G.* ‘Lilac Ice’ ^x^	-	-	-	*G. wallichianum* (W)	**6.78–6.86**	**28**, 26, 28, 56	**0.242–0.245**	*G.* ‘Rozanne’ (W × PR)	**9.25**	**42**	**0.220**
G13	*G.* × *riversleaianum* ‘Mavis Simpson’ ^x^	**4.31 (p)**	**38**	**0.113**	*G. endressii* (E)	**3.12–3.48**, 3.13	**26/28**, 26, 28	**0.120–0.124**	*G. traversii* (SE)	-	28, 52, 54, 56	-
G15	*G.* ‘Patricia’ ^x^	**5.10 (P)**	-	-	*G. endressii* (E)	**3.12–3.48**, 3.13	**26/28**, 26, 28	**0.120– 0.124**	*G. psilostemon* (SY)	**5.18**	**42**	**0.123**
G16	*G.* ‘Rozanne’ ^x^	**9.25 (M)**	**42**	**0.220**	*G. wallichianum* (W)	**6.78–6.86**	**28**, 26, 28, 56	**0.242–0.245**	*G. himalayense* (PR)	**7.31–7.49**	**56**	**0.130–0.134**
G18	*G.* ‘Sanne’ ^x^	**4.07 (M)**	**38**	**0.107**	*G. sessiliflorum* (SE)	-	**26**, 28, 52	-	*G.* × *oxonianum* (E)	**3.22–3.73**	**26**	**0.124–0.143**
G22	*G.* ‘Tanya Rendall’ ^w^	**3.91 (M)**	**38**	**0.103**	*G.* × *antipodeum*^y^ (SE)	-	-	-	*G.* × *oxonianum* (E)	**3.22–3.73**	**26**	**0.124–0.143**
G24	*G.* ‘Tiny Monster’ ^w^	**12.94 (P)**	**108/110**	**0.117–0.120**	*G. sanguineum* (SA)	**8.78**, 8.36, 8.38	**84**, 84	**0.104**	*G. psilostemon* (SY)	**5.18**	**42**	**0.123**
G75	*G.* ‘Bloomtime’ ^v^	**6.86 (M)**	**26/28**	**0.245–0.264**	*G. wallichianum* (W)	**6.78–6.86**	**28**, 26, 28, 56	**0.242–0.245**	*G. himalayense* (PR)	**7.31–7.49**	**56**	**0.130–0.134**
G80	*G.* ‘Blushing Turtle’ ^x^	**7.80 (M)**	**66**	**0.118**	*G. sanguineum* (SA)	**8.78**, 8.36, 8.38	**84**, 84	**0.104**	*G.*× *oxonianum* (E) or *G. sphodeloides* (Dissecta)	**3.22–3.73** or -	**26** or 24, 26, 28	**0.124–0.143** or -
**Subgenus *Robertium-*Intragroup Crosses**												
**Genotype Number**	**Name**	**Gen. Size (pg/2C)**	**Chrom. numb. (2n = )**	**DNA/chrom.**	**Parent 1**	**Gen. Size (pg/2C)**	**Chrom. numb. (2n = )**	**DNA/chrom.**	**Parent 2**	**Gen. Size (pg/2C)**	**Chrom. numb. (2n = )**	**DNA/chrom.**
G26 ^z^	*G.* × *cantabrigiense* ‘Berggarten’ ^w^	-	-	-	*G. maccrorhizum* (MA)	**1.60**, 1.49, 2.89, 3.37, 3.71	**46/92**, 46, 92	-	*G. dalmaticum* (MA)	**1.56**, 1.44	46	**0.033**
G27	*G.* × *cantabrigiense* ‘Biokovo’ ^x^	**1.51 (M)**	**46**	**0.033**	*G. maccrorhizum* (MA)	**1.60**, 1.49, 2.89, 3.37, 3.71	**46/92**, 46, 92	**0.035**	*G. dalmaticum* (MA)	**1.56**, 1.44	46	**0.033**
G28 ^z^	*G.* × *cantabrigiense* ‘Karmina’ ^w^	-	-	-	*G. maccrorhizum* (MA)	**1.60**, 1.49, 2.89, 3.37, 3.71	**46/92**, 46, 92	-	*G. dalmaticum* (MA)	**1.56**, 1.44	46	**0.033**
G29 ^z^	*G.* × *cantabrigiense* ‘St Ola’ ^w^	-	-	-	*G. maccrorhizum* (MA)	**1.60**, 1.49, 2.89, 3.37, 3.71	**46/92**, 46, 92	-	*G. dalmaticum* (MA)	**1.56**, 1.44	46	**0.033**

^z^ These genotypes are not further characterized for genome sizes and chromosome numbers. ^y^ *G.* x *antipodeum* is a cross between *G. sessiliflorum* and *G. traversii*. Source of plant material with ^x^ Denis-Plants, ^w^ Jan Spruyt—Van der Jeugd, ^v^ Kwekerij Jan Neelen.

**Table 3:** Calculation of the pairwise genetic proximity measures (Jaccard similarity coefficients) of some different *Geranium* species and cultivars used in obtained cross combinations. When more than one species was available, a mean value was used and designated by *. The numbers depicted in orange are intergroup cross combinations; numbers in blue are intragroup crosses.

Genotype	1	2	3 *	4 *	5	6	7	8 *	9	10 *	11 *	12 *	13 *	14 *	15 *	16	17 *	18	19
1. *G.* ‘Brookside’	1.000																		
2. *G. pratense*	0.298	1.000																	
3. G. cinereum *	0.132	0.120	1.000																
4. G. psilostemon *	0.201	0.178	0.163	1.000															
5. *G.* ‘Rozanne’	0.273	0.310	0.127	0.186	1.000														
6. *G.* ‘Salome’	0.180	0.185	0.143	0.183	0.189	1.000													
7. *G. nodosum*	0.209	0.173	0.146	0.181	0.185	0.165	1.000												
8. G. wallichianum *	0.194	0.220	0.134	0.188	0.426	0.179	0.204	1.000											
9. *G.* × *cantabrigiense*	0.136	0.091	0.098	0.115	0.105	0.131	0.101	0.105	1.000										
10. G. endressii *	0.146	0.155	0.133	0.292	0.147	0.151	0.173	0.136	0.151	1.000									
11. G. himalayense *	0.348	0.302	0.156	0.235	0.259	0.194	0.208	0.181	0.115	0.173	1.000								
12. G. macrorrhizum *	0.135	0.091	0.099	0.127	0.108	0.117	0.115	0.106	0.316	0.152	0.115	1.000							
13. G. maculatum *	0.150	0.159	0.164	0.162	0.143	0.162	0.125	0.127	0.113	0.166	0.173	0.106	1.000						
14. *G.* × oxonianum *	0.153	0.141	0.124	0.244	0.143	0.130	0.152	0.143	0.128	0.593	0.161	0.148	0.147	1.000					
15. G. phaeum *	0.125	0.136	0.165	0.135	0.137	0.129	0.129	0.149	0.111	0.119	0.137	0.094	0.111	0.135	1.000				
16. *G. renardii*	0.124	0.111	0.146	0.128	0.124	0.153	0.153	0.137	0.108	0.132	0.126	0.109	0.148	0.127	0.146	1.000			
17. G. sanguineum *	0.193	0.169	0.132	0.167	0.178	0.171	0.194	0.160	0.146	0.150	0.198	0.144	0.144	0.139	0.147	0.144	1.000		
18. *G. sylvaticum*	0.201	0.204	0.135	0.192	0.165	0.148	0.175	0.172	0.121	0.149	0.189	0.120	0.149	0.150	0.156	0.123	0.196	1.000	
19. *G. versicolor*	0.152	0.135	0.131	0.281	0.121	0.142	0.142	0.153	0.144	0.506	0.156	0.136	0.149	0.484	0.128	0.136	0.153	0.147	1.000
20. *G. wlassovianum*	0.163	0.151	0.143	0.177	0.186	0.159	0.240	0.217	0.134	0.128	0.216	0.126	0.137	0.152	0.145	0.152	0.156	0.203	0.135

## Data Availability

The data presented in this study are available on request from the corresponding author. The data are not publicly available because this research was in collaboration with a private company.

## Supplementary Materials

The following are available online at https://www.mdpi.com/article/10.3390/genes12050730/s1, Figure S1: Classification of *Geranium* genus according to the Yeo, Figure S2: Phylogenetic tree of 45 *Geranium* genotypes with their genome sizes (pg/2C), chromosome numbers (Chrom. numb. (2n)) and calculated DNA content per chromosome (DNA/chromo. (pg/chromosome)), Figure S3: (a) Genome sizes (pg/2C), (b) chromosome numbers (2n) and (c) calculated DNA content per chromosome (pg/chromosome) of different genotypes of *Geranium* divided into taxonomic groups according to Yeo.
