# Supplementary material for: Can Knowledge of Genetic Distances, Genome Sizes and Chromosome Numbers Support Breeding Programs in Hardy Geraniums?
Source: Genes (Basel). 2021 May 13;12(5):730. doi: 10.3390/genes12050730 (PMC8152959; doi:10.3390/genes12050730)
Supplement: Supplementary file 1 [file genes-12-00730-s001.zip › genes-1185028-supplementary.pdf]

- *Geranium* Genus
  - 1. Subgenus *Geranium*
    - Section Sylvaticum
      - Endressii Group
        - *G. Endressii*
        - *G.* x *oxonianum*
        - *G.* x *riversleaianum*
        - *G. versicolor*
        - *G. nodosum*
      - Sylvaticum Group
        - *G. sylvaticum*
        - *G. psilastemon*
      - Pratense Group
        - *G. pratense*
        - *G. himalayense*
        - *G. clarkei*
      - Wallichianum Group
        - *G. wallichianum*
        - *G. lambertii*
        - *G. procurrens*
      - Palustre Group
        - *G. wlassovianum*
      - Sanguineum Group
        - *G. sanguineum*
      - Sessiliflorum Group
        - *G. traversii*
        - *G.* x *antipodeum*
        - *G. sessiliflorum*
      - Maculatum Group
        - *G. maculatum*
    - Section Tuberosum
      - Platypetalum Group
        - *G. renardii*
  - 2. Subgenus *Robertium*
    - Section Unguiculata
      - Macrorrhizum Group
        - *G. maccrorhizum*
        - *G.* x *cantabrigiense*
        - *G. dalmaticum*
  - 3. Subgenus *Erodioidea*
    - Section Erodioidea
      - Phaeum Group
        - *G. phaeum*
    - Section Subacaulia
      - Cinereum Group
        - *G. cinereum*

Figure S1. Classification of *Geranium* genus according to the Yeo [7].

2C-value (pg) + reference	Chrom. numb. (2n)	DNA/chrom. (pg/chromosome)	Subgenus	Genotype	
8.90 (M)	42	0.212	GE	*G.* 'Azure Rush' [W x (W x PR)]	G02
9.25 (M)	42	0.220	GE	*G.* 'Rozanne' [W x PR]	G16
-	-	-	GE	*G.* 'Lilac Ice' [W x (W x PR)]	G11
6.86 (M)	26/28	0.245 – 0.264	GE	*G.* 'Bloomtime' [W x PR]	G75
6.86 (P)	28	0.245	GE	*G. wallichianum* 'Havana Blue' [W]	G76
6.78 (P)	28	0.242	GE	*G. wallichianum* 'Sylvia's Surprise' [W]	G21
4.92 (P)	28	0.175	GE	*G. pratense* 'Galactic' [PR]	G09
5.08 (P)	28	0.185	GE	*G. pratense* 'Algera Double' [PR]	G57
5.08 (P)	28	0.181	GE	*G. pratense* 'Purple Ghost' [PR]	G61
4.54 (M)	28	0.162	GE	*G.* 'Brookside' [PR]	G04
6.93 (P)	42	0.165	GE	*G.* 'Orion' [PR]	G14
7.31 (M)	56	0.130	GE	*G. himalayense* 'Baby Blue' [PR]	G38
7.49 (M)	56	0.134	GE	*G. himalayense* 'Derrick Cook' [PR]	G39
3.38 (M)	28	0.121	GE	*G. nodosum* 'Silverwood' [E]	G19
11.5 (P)	56	0.205	GE	*G. wlassovianum* [PA]	G77
4.76 (P)	28	0.170	GE	*G. sylvaticum* 'Album' [SY]	G71
12.94 (P)	108/110	0.117 - 0.120	GE	*G.* 'Tiny Monster' [SA x SY]	G24
7.80 (M)	66	0.118	GE	*G.* 'Blushing Turtle' [SA x E or SA x Dissecta]	G80
8.78 (M)	84	0.104	GE	*G. sanguineum* 'Album' [SA]	G69
8.27 (M)	58	0.143	GE	*G.* 'Anne Thomson' [W x SY]	G01
12.57 (P)	88	0.143	GE	*G.* 'Dragon Heart' [W x SY]	G07
5.10 (P)	-	-	GE	*G.* 'Patricia' [SY x E]	G15
5.18 (P)	42	0.123	GE	*G. psilostemon* [SY]	G62
6.95 (M)	54	0.129	GE	*G.* 'Catherine Deneuve' [SY x E or SY x W]	G05
3.12 (M)	26	0.120	GE	*G. endressii* [E]	G35
3.22 (P)	26	0.124	GE	*G.* x *oxonianum* 'Southcombe Double' [E]	G50
3.48 (M)	28	0.124	GE	*G. endressii* 'Trevor Bath' [E]	G37
3.72 (M)	26	0.143	GE	*G.* x *oxonianum* 'Katherine Adele' [E]	G49
3.53 (M)	26/28	0.126 - 0.136	GE	*G. versicolor* [E]	G73
4.07 (M)	38	0.107	GE	*G.* 'Sanne' [SE x E]	G18
3.91 (M)	38	0.103	GE	*G.* 'Tanya Rendall' [SE x E]	G22
-	-	-	GE	*G.* 'Dusky Crûg' [SE x E]	G08
4.31 (P)	38	0.113	GE	*G.* x *riversleaianum* 'Mavis Simpson' [SE x E]	G13
5.31 (S)	38	0.140	GE	*G.* 'Bob's Blunder' [Unknown]	G03
9.25 (S)	-	-	GE	*G.* 'Salome' [W]	G17
2.31 (M)	28	0.082	GE	*G.* 'Chantilly' [PL]	G06
2.35 (M)	28	0.084	GE	*G. renardii* [PL]	G64
7.64 (M)	52	0.147	GE	*G. maculatum* 'Album' [M]	G45
7.63 (M)	52	0.147	GE	*G. maculatum* 'Elizabeth Ann' [M]	G46
6.39 (P)	-	-	ER	*G. cinereum* 'Jolly Jewel Red' [C]	G10
6.06 (P)	28	0.217	ER	*G. cinereum* 'Laurence Flatman' [C]	G30
3.52 (M)	28	0.126	ER	*G. phaeum* 'Angelina' [P]	G54
1.51 (M)	46	0.033	RO	*G.* x *cantabrigiense* 'Biokovo' [MA]	G27
1.60 (M)	46	0.035	RO	*G. macrorrhizum* 'Czakor' [MA]	G42
2.96 (M)	92	0.032	RO	*G. macrorrhizum* 'White Ness' [MA]	G44

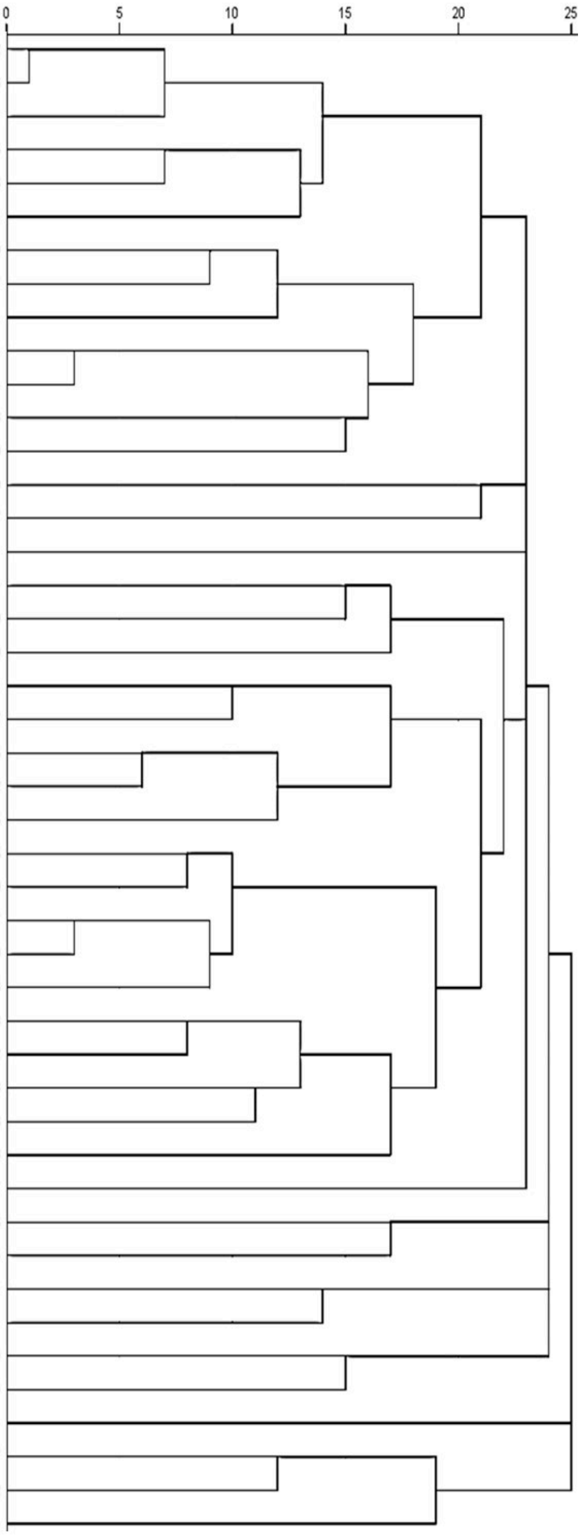

Figure S2. Phylogenetic tree of 45 *Geranium* genotypes with their genome sizes (pg/2C), chromosome numbers (Chrom. numb. (2n)) and calculated DNA content per chromosome (DNA/chromo. (pg/chromosome)). The standard deviation of the genome sizes was always equal to or below 0.09. The plant species used as internal standard is mentioned with the genome size (*Zea mays* (M), *Pisum sativum* (P) and *Glycine max* (S)). The different subgenera are noted by GE (*Geranium*), ER (*Erodidoidea*) and RO (*Robertium*). The abbreviation between brackets after the plant genotype refers to the taxonomic group according to Yeo [7]. For abbreviations see Table 1 and Table 2.

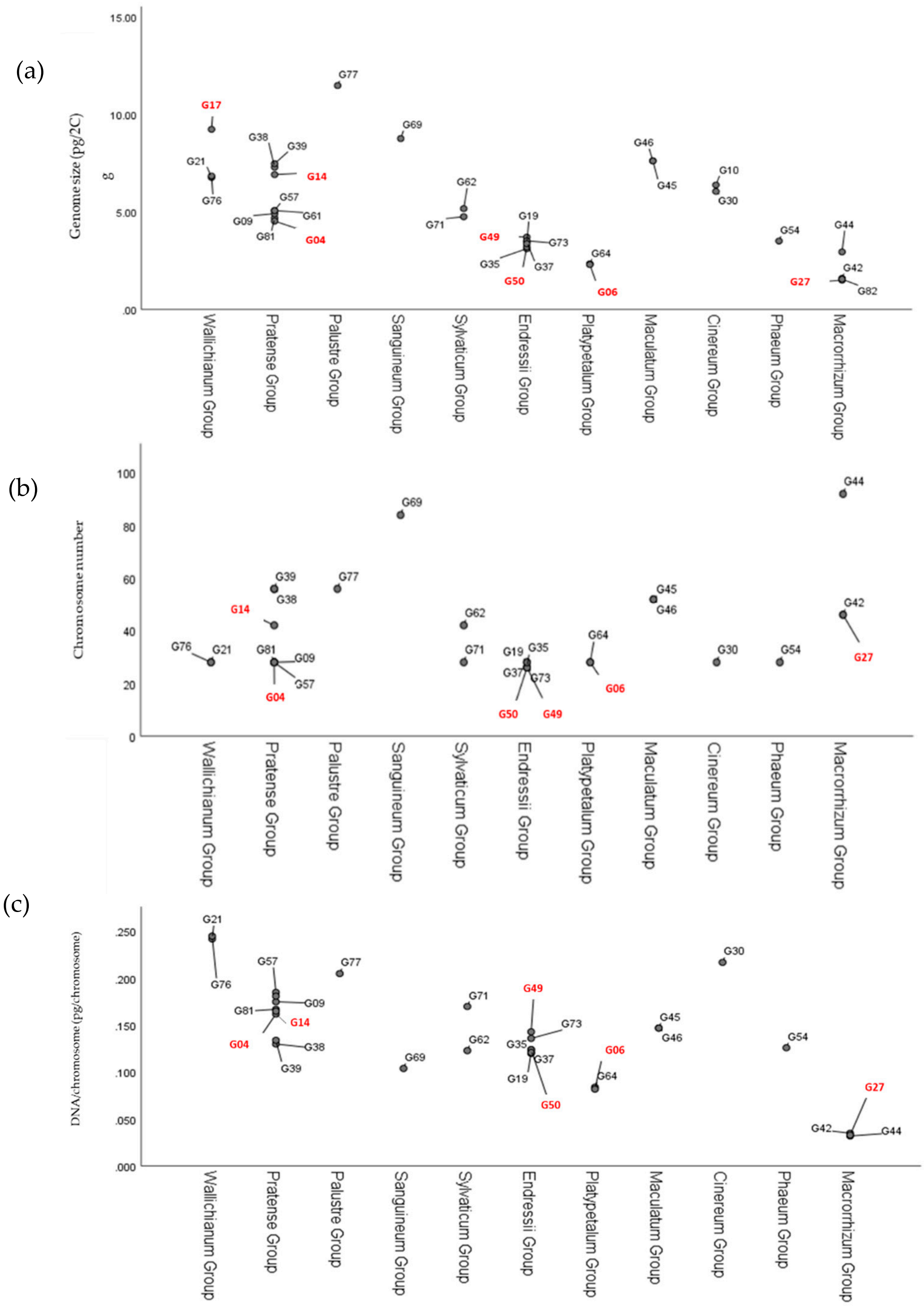

Figure S3. (**a**) Genome sizes (pg/2C), (**b**) chromosome numbers (2n) and (**c**) calculated DNA content per chromosome (pg/chromosome) of different genotypes of *Geranium* divided into taxonomic groups according to Yeo [7] (intragroup hybrids are included in red; intergroup hybrids not included).
